# Nutrients, minerals, antioxidant pigments and phytochemicals, and antioxidant capacity of the leaves of stem amaranth

**DOI:** 10.1038/s41598-020-60252-7

**Published:** 2020-03-03

**Authors:** Umakanta Sarker, Shinya Oba, Moses Ahmed Daramy

**Affiliations:** 1 Department of Genetics and Plant Breeding, Faculty of Agriculture, Bangabandhu Sheikh Mujibur Rahman Agricultural University, Gazipur, 1706 Bangladesh; 2 Laboratory of Field Science, Faculty of Applied Biological Sciences, Gifu University, Yanagido 1-1 Gifu, Japan; 3 Soil, Crop and Natural Resource Management, Sierra Leone Agricultural Research Institute, Tower Hill Freetown Sierra Leone, PMB 1313 Sierra Leone

**Keywords:** Biochemistry, Plant sciences

## Abstract

We evaluated 17 genotypes of stem amaranth (*Amaranthus lividus*) in terms of dietary fiber, moisture, carbohydrates, fat, ash, gross energy, protein, minerals, phytopigments, total antioxidant capacity (TAC), vitamins, total flavonoids (TFC), total polyphenols (TPC) and their variations. Stem amaranth leaves have abundant dietary fiber, moisture, carbohydrates, and protein. We found significant amount of potassium, calcium, magnesium (9.61, 24.40, and 29.77 mg g^−1^ DW), iron, manganese, copper, zinc, (1131.98, 269.89, 25.03, and 1006.53 µg g^−1^ DW), phytopigments such as chlorophyll *a*, chlorophyll *ab* chlorophyll *b*, (27.76, 42.06, and 14.30 mg 100 g^−1^ FW), betalain, betaxanthin, betacyanin (62.92, 31.81, 31.12 µg 100 g^−1^ FW), total carotenoids, beta-carotene (1675.38, 1289.26 µg g^−1^ FW), vitamin C (1355.46 µg g^−1^ FW), TPC, TFC (228.63 GAE and 157.42 RE µg g^−1^ DW), and TAC (DPPH, ABTS^+^) (26.61, 51.73 TEAC µg g^−1^ DW) in the leaves of stem amaranth. Genotypes exhibited a wide range of variations. Three genotypes DS40, DS30, and DS26 could be used as an antioxidant profile enriched stem amaranth. Phenolics, phytopigments, flavonoids, and vitamins of stem amaranth leaves exhibited strong antioxidant activity. Stem amaranth could be a potential source of dietary fiber, moisture, carbohydrates, protein, minerals, phenolics, phytopigments, flavonoids, and vitamins in our daily diet for attaining nutritional and antioxidant sufficiency.

## Introduction

Amaranth has great variability and phenotypic plasticity^1^ with many culinary uses. In Bangladesh including south-east Asia, Africa, South America, the edible stem amaranth leaves are a very famous vegetable. Its popularity is continuously increasing in the Asian continent and elsewhere because of high nutritional value, taste, and attractive leaf color. In Bangladesh, stem amaranth is grown year-round and it could be grown in the gaps period of leafy vegetables between winter and hot summer^2,3^. It is an inexpensive vegetable and has abundant dietary fiber and protein with essential amino acids such as methionine and lysine, minerals, pigments and phytochemicals like betacyanin, betaxanthin, chlorophyll, carotenoids, beta-carotene, vitamin C, phenolic compounds, and flavonoids^4–10^.

In the world, food insecurity results in a continuous calorie deficit of approximately 795 million malnourished people^11^. Deficiency of vitamins or minerals results in hidden hunger in over two billion people^12^. Staple foods are deficient of micronutrients, mainly iron, zinc and iodine, pro-vitamin A, carotenoids, vitamin C, E, albeit these are a source of energy^13^. Consequently, staple foods in our daily diet result in hidden hunger^12^. We can ensure a balanced and healthy diet by consumption of fruit and vegetables as a source of vitamins and minerals accomplished with staple food. Furthermore, we protect human health and reduce the risk of cancer, cardiovascular, and other chronic diseases by feeding fruit and vegetables. Phytochemical compounds such as leaf pigments, vitamin C, phenolic and flavonoids are thought to contribute to those health benefits^14–16^.

Recently, natural antioxidants of vegetables attracted consumers and researchers. Leaf pigments (betacyanin, betaxanthin, chlorophyll, and carotenoids), vitamin C, phenolics and flavonoids are available natural antioxidants in amaranths^4,17^. These natural antioxidants phytochemicals defense against several diseases like cardiovascular diseases, cancer, cataracts, atherosclerosis, retinopathy, arthritis, emphysema, and neurodegenerative diseases^17–19^. Amaranth is also tolerant to abiotic stresses like drought and salinity^20–24^.

Stem amaranth is a very popular vegetable in Bangladesh. It is consumed both as a leafy vegetable in early stages and vegetables (stem only) in the later stage. In the younger stage, around 30 days old, the whole plant including leaves and tender succulent stems are used as leafy vegetables. The large barreled stem of this amaranth is succulent and juicy and become edible as vegetables up to initiation of flowering. It takes approximately two to three months to flower, even though some photosensitive cultivar takes 9 to 12 months to flower. Those large barreled juicy and succulent stems are a famous vegetable in Bangladesh and consumed year-round. However, the literature has shown that amaranth leaf had much higher nutrients, minerals, pigments, phytochemicals, and antioxidants in comparison to the stem of the plant^4,25^. For this reason, we evaluated the stem amaranth as leafy vegetables in terms of nutrients, minerals, antioxidant pigments and phytochemicals, and antioxidant capacity. Although it is abiotic stress tolerant and inexpensive sources of minerals, dietary fiber, protein, and antioxidant phytochemicals like leaf pigments, vitamin C, phenolics, and flavonoids, there is a scarce of information in this species. In our earlier study, we evaluated *A. tricolor* for morphological, proximate, minerals, antioxidant leaf pigments, antioxidant phytochemicals^2,3,5–10^. To our knowledge, it is the first report on proximate and mineral compositions, phenolics, flavonoids, leaf pigments, and vitamins in a huge number of diversified stem amaranth germplasms available in Bangladesh and elsewhere. Therefore, to fill these gaps, the present investigation was undertaken to evaluate proximate and mineral compositions, leaf pigments, vitamins, phenolics, and flavonoids content in 17 stem amaranth genotypes. To determine the variability of these traits in 17 stem amaranth genotypes.

## Results and Discussion

### Proximate compositions

Table 1 represents the proximate compositions of stem amaranth. The leaf water content ranged from 82.05 to 88.43 g 100 g^−1^ FW. As high leaf dry matter obtained from lower moisture contents, five genotypes (17–18% dry matter) had a considerable dry matter. The maturity of the plant directly associated with the leaf moisture content of stem amaranth. The findings obtained in this study were fully agreed to the reports of amaranth and sweet potato leaves by Sarker and Oba^26^ and Sun *et al*.^27^, respectively.

**Table 1:** Proximate compositions (per 100 g fresh weight) and dietary fiber (µg g^−1^ FW) of 17 stem amaranth genotypes.

Genotypes	Moisture (g)	Protein (g)	Fat (g)	Carbohydrates (g)	Energy (Kcal)	Ash (g)	Dietary fiber (µg g^−1^ FW)
DS25	86.45 ± 0.98c	4.20 ± 0.02e	0.42 ± 0.01a	5.81 ± 0.06l	41.02 ± 0.34n	3.12 ± 0.02h	85.74 ± 0.95c
DS26	82.15 ± 0.88g	5.38 ± 0.03c	0.21 ± 0.01f	7.11 ± 0.10j	52.99 ± 0.48b	5.15 ± 0.01b	78.21 ± 0.75h
DS27	83.74 ± 1.71f	1.47 ± 0.03n	0.28 ± 0.01d	9.85 ± 0.12a	46.61 ± 0.82d	4.66 ± 0.01c	83.56 ± 0.85d
DS28	85.66 ± 2.41e	3.53 ± 0.03f	0.27 ± 0.03d	8.26 ± 0.16e	46.23 ± 0.76f	2.28 ± 0.02l	83.85 ± 0.41d
DS29	85.55 ± 1.83e	3.22 ± 0.03g	0.24 ± 0.03e	8.07 ± 0.11f	43.85 ± 0.88h	2.92 ± 0.06j	77.46 ± 0.46i
DS30	82.05 ± 1.26g	5.16 ± 0.05d	0.24 ± 0.04e	7.12 ± 0.21j	53.38 ± 0.46a	5.43 ± 0.04a	82.75 ± 0.77e
DS31	86.26 ± 1.11d	2.25 ± 0.04k	0.22 ± 0.03f	8.03 ± 0.08f	41.22 ± 0.43l	3.24 ± 0.03g	73.82 ± 0.47k
DS32	85.41 ± 1.18e	3.56 ± 0.05f	0.35 ± 0.02b	7.60 ± 0.10h	48.17 ± 0.82c	3.08 ± 0.02i	79.41 ± 0.65f
DS33	85.77 ± 1.44e	2.57 ± 0.05h	0.36 ± 0.02b	7.38 ± 0.13i	42.64 ± 0.56i	3.92 ± 0.03e	62.40 ± 0.46o
DS34	88.43 ± 1.03a	5.56 ± 0.04b	0.35 ± 0.03b	2.21 ± 0.10n	35.91 ± 0.48q	3.45 ± 0.05f	74.54 ± 0.74j
DS35	85.45 ± 1.15e	2.38 ± 0.04j	0.27 ± 0.02d	8.46 ± 0.05d	41.72 ± 0.43k	3.44 ± 0.05f	78.73 ± 0.48g
DS36	83.57 ± 1.31f	5.76 ± 0.03a	0.28 ± 0.01d	5.73 ± 0.15l	46.45 ± 0.49e	4.66 ± 0.04c	72.87 ± 0.48l
DS37	87.52 ± 1.49b	1.87 ± 0.01l	0.32 ± 0.03c	7.84 ± 0.15g	37.74 ± 0.51o	2.45 ± 0.05k	95.72 ± 0.52a
DS38	83.55 ± 1.58f	2.49 ± 0.05i	0.22 ± 0.03f	9.68 ± 0.15b	44.07 ± 0.46g	4.06 ± 0.03d	66.54 ± 0.27n
DS39	86.75 ± 1.46c	4.24 ± 0.03e	0.43 ± 0.02a	5.13 ± 0.11m	41.95 ± 0.47j	3.45 ± 0.02f	89.52 ± 0.42b
DS40	86.55 ± 1.57c	3.57 ± 0.06f	0.22 ± 0.02f	6.21 ± 0.14k	37.08 ± 0.52p	3.45 ± 0.02f	83.76 ± 0.41d
DS41	87.38 ± 1.24b	1.57 ± 0.02m	0.31 ± 0.02c	8.65 ± 0.15c	41.13 ± 0.34m	2.09 ± 0.01m	72.28 ± 0.56m
Mean	85.43	3.46	0.29	7.24	43.66	3.58	78.89
CV%	1.6258	0.3542	0.1284	0.1675	0.3245	0.5365	0.6345

CV, Coefficient of variation; n = 6; **Significant at 1% level, Different letters in each columns are differed significantly by Tukey’s HSD test.

The protein content of the leaf of stem amaranth exerted much pronounced variations. The protein content ranged from 5.76 to 1.47 g 100 g^−1^ FW. Nine genotypes had higher protein content compared to their average values. As leafy vegetables, the genotype DS36, DS34, DS26, DS30, DS25, and DS39 had high protein content. Stem amaranth is the main source of protein for poor people of the low-income countries and vegetarians. Our results showed that stem amaranth exhibited high protein content (3.46 g 100 g^−1^ FW) than *A. tricolor* (1.26%) of our previous study^2^.

The fat of stem amaranth ranged from 0.43, 0.42 to 0.21 g 100 g^−1^ FW with a grand mean value of 0.29 g 100 g^−1^ FW, and showing the following order: DS33 > DS32 > DS34 > DS37 > DS41. Sarker and Oba^26^ and Sun *et al*.^27^ observed similar results in *A. tricolor* and the leaf of sweet potato, respectively, They reported that cell function, body temperature, and the insulation of body organs were maintained through catabolism of fat. Fats are an excellent source of omega-6 and omega-3 fatty acids. Absorption, digestion, and transport of fat-soluble vitamins such as A, D, E, and K mainly depend on fats. The carbohydrates content ranged from 9.85 to 2.21 g 100 g^−1^ FW with a mean value of 7.24 g 100 g^−1^ FW. The energy ranged from 53.38 to 35.91 Kcal 100 g^−1^ FW with a grand mean value of 43.66 Kcal 100 g^−1^ FW. Ash content ranged from 5.43 to 2.09 g 100 g^−1^ FW with a grand mean value of 3.58 g 100 g^−1^ FW.

The significant variations were observed in 17 stem amaranth genotypes in terms of dietary fiber. Dietary fiber ranged from 95.72 to 62.40 µg g^−1^ FW with a mean value of 78.89 µg g^−1^ FW. Dietary fiber significantly contributed to the cure of constipation, digestibility, and palatability^6^. Our results showed that the leaf of stem amaranth were a good source of dietary fiber, moisture, carbohydrates, and protein. The results of this study corroborated with the results of Sarker and oba^26^.

### Composition of minerals

Table 2 represents the content of minerals of stem amaranth. In this study, the content of potassium (K) varied from 6.54 mg g^−1^ to 14.21 mg g^−1^ DW. High potassium content was obtained from eight genotypes with a grand mean value of 9.61 mg g^−1^ DW. The potassium content of ten genotypes was much higher than their grand mean. The range of Ca content was 16.06–31.22 mg g^−1^ DW. High Ca content was noted in eight genotypes which were better than the respective average value. Mg content did not exhibit pronounced variations in 17 stem amaranth genotypes (27.71 to 32.53 mg g^−1^ DW). The average Mg content was 29.77 mg g^−1^ DW. High Mg content was noted in three genotypes. In our present study, we found a significant amount of K (9.61 mg g^−1^), calcium (24.40 mg g^−1^) and magnesium (29.77 mg g^−1^) in the leaf of stem amaranth, albeit we determined based on the dry weight. Chakrabarty *et al*.^28^ in stem amaranth and Sarker and Oba^26^ in *A. tricolor* also observed similar results. Jimenez-Aguiar and Grusak^29^ reported a good amount of Mg, K, and Ca in different species of amaranth. They reported that Mg, Ca, and K content of different species of amaranth was much higher than kale, black nightshade, spider flower, and spinach.

**Table 2:** Mineral compositions (Macroelements mg g^−1^ DW and microelements µg g^−1^ DW elements) of 17 stem amaranth genotypes.

Genotypes	Macroelements (mg g^−1^ DW)			Microelements (µg g^−1^ DW)			
	K	Ca	Mg	Fe	Mn	Cu	Zn
DS25	7.34 ± 0.02f	16.24 ± 0.05j	29.97 ± 0.07c	1047.74 ± 0.86g	228.28 ± 0.27j	26.32 ± 0.04d	852.24 ± 0.74o
DS26	14.43 ± 0.06a	17.94 ± 0.05i	31.88 ± 0.12a	1732.94 ± 0.56b	345.34 ± 0.46b	23.56 ± 0.06g	1534.56 ± 0.51a
DS27	9.85 ± 0.07d	25.67 ± 0.04e	29.32 ± 0.14f	989.67 ± 0.87i	198.72 ± 0.39k	20.68 ± 0.04i	914.88 ± 0.46l
DS28	7.52 ± 0.04f	25.66 ± 0.05e	29.86 ± 0.16d	986.69 ± 0.76j	188.76 ± 0.28l	20.73 ± 0.04i	941.74 ± 0.64k
DS29	11.55 ± 0.05c	24.23 ± 0.06f	29.55 ± 0.14e	1033.56 ± 0.48h	272.27 ± 0.57f	28.17 ± 0.07c	944.42 ± 0.51j
DS30	10.34 ± 0.05d	31.32 ± 0.08a	30.23 ± 0.18d	1116.91 ± 0.34e	321.83 ± 0.37c	27.95 ± 0.07c	1432.27 ± 0.41b
DS31	9.98 ± 0.04d	29.65 ± 0.06c	29.22 ± 0.17f	1384.65 ± 0.62c	381.26 ± 0.64a	18.14 ± 0.04j	1241.35 ± 0.37c
DS32	8.36 ± 0.06e	30.46 ± 0.06b	30.84 ± 0.14b	2572.22 ± 0.46a	310.87 ± 0.68d	25.34 ± 0.04e	1023.28 ± 0.46e
DS33	11.37 ± 0.07c	28.25 ± 0.05d	30.24 ± 0.16d	968.42 ± 0.61k	312.65 ± 0.53d	29.33 ± 0.03b	988.33 ± 0.34g
DS34	12.41 ± 0.06b	19.34 ± 0.07h	29.89 ± 0.15d	752.23 ± 0.42n	176.84 ± 0.45m	44.42 ± 0.04a	748.47 ± 0.48p
DS35	6.62 ± 0.06g	24.21 ± 0.05f	29.32 ± 0.09f	985.65 ± 0.82j	246.72 ± 0.81h	28.46 ± 0.06c	957.18 ± 0.29i
DS36	10.06 ± 0.07d	28.78 ± 0.04d	29.82 ± 0.14d	1128.56 ± 0.48e	271.55 ± 0.68f	24.78 ± 0.04f	1052.33 ± 0.48d
DS37	12.16 ± 0.08b	19.28 ± 0.05h	28.68 ± 0.15g	743.12 ± 0.15o	296.76 ± 0.66e	24.87 ± 0.02f	1005.32 ± 0.68f
DS38	6.63 ± 0.04g	24.13 ± 0.07f	29.56 ± 0.17e	788.43 ± 0.54m	239.54 ± 0.38i	27.85 ± 0.06c	889.38 ± 0.57m
DS39	7.37 ± 0.06f	22.79 ± 0.05g	27.76 ± 0.12h	1135.29 ± 0.62d	251.31 ± 0.61g	23.54 ± 0.07g	976.87 ± 0.45h
DS40	11.54 ± 0.04c	24.86 ± 0.07e	30.58 ± 0.16c	1062.84 ± 0.52f	276.67 ± 0.85f	22.32 ± 0.03h	878.46 ± 0.51n
DS41	7.64 ± 0.05f	23.26 ± 0.07fg	28.71 ± 0.15g	932.25 ± 0.38l	337.21 ± 0.53b	25.36 ± 0.03e	901.38 ± 0.27l
Mean	9.72	24.47	29.73	1138.89	273.92	25.99	1016.62
CV%	2.876	1.352	1.754	0.528	0.645	0.543	0.462

CV, Coefficient of variation; K, Potassium; Ca. Calcium, Mg, Magnesium; Fe, Iron; Mn, Manganese; Cu, Copper; Zn, Zinc; n = 6; **Significant at 1% level, Different letters in each columns are differed significantly by Tukey’s HSD test.

Iron content showed the prominent variations in terms of genotypes (739.04 µg g^−1^ DW to 2546.25 µg g^−1^ DW). The grand mean value of 17 genotypes was 1131.98 µg g^−1^ DW. High iron content was obtained from four genotypes which were higher than the mean value. The range of manganese content varied from 174.63 µg g^−1^ DW to 375.33 µg g^−1^ DW, with a mean value of 269.89 µg g^−1^ DW. Six genotypes had high manganese content. The significant and notable variations in copper content were reported in the genotypes studied (17.56–42.15 µg g^−1^ DW). High copper was obtained from eight genotypes which were higher than the mean value. The zinc content of stem amaranth varied significantly in terms of genotypes (741.50 µg g^−1^ DW to 1525.92 µg g^−1^ DW). High zinc content was observed in five genotypes which were higher than the grand mean value (1006.53 µg g^−1^ DW). Stem amaranth leaves contained higher zinc and iron content than the cassava leaves^30^ and beach pea^31^. Our study showed that leaves of stem amaranth had considerable iron (1131.98 µg g^−1^), manganese (269.89 µg g^−1^), copper (25.03 µg g^−1^), and zinc (1006.53 µg g^−1^), albeit it was measured based on the dry weight. Jimenez-Aguiar and Grusak^29^ reported a good amount of iron, manganese, copper, and zinc in the different species of amaranth. They reported that iron, manganese, copper, and zinc content of different species of amaranth were much higher than kale, black nightshade, spider flower, and spinach.

### Composition of antioxidant leaf pigments

Table 3 represents the composition of antioxidant leaf pigments of stem amaranth. chlorophyll *a* content differed remarkably in stem amaranth (12.25 to 50.86 mg 100 g^−1^). Chlorophyll *a* content was high in three stem amaranth genotypes. Chlorophyll *a* content of seven genotypes was higher than the average value. There were prominent variations in chlorophyll *b* content of 17 stem amaranth genotypes (5.67 to 27.38 mg 100 g^−1^). Prominent variations were also observed in chlorophyll *ab* (18.86 to 74.37 mg 100 g^−1^). Four genotypes exhibited high chlorophyll *ab* content, Nine genotypes had higher chlorophyll *ab* than the mean value. Our study revealed that stem amaranth genotypes had a considerable amount of chlorophyll *ab* (42.06 mg 100 g^−1^), chlorophyll *a* (27.76 mg 100 g^−1^), and chlorophyll *b* (14.30 mg 100 g^−1^), whereas, chlorophylls content of *A. tricolor* reported by Khanam and Oba^32^ were relatively lower.

**Table 3:** Mean performance for antioxidant leaf pigments in 17 stem amaranth genotypes.

Genotypes	chlorophyll *a* (mg 100 g^−1^ FW)	Chlorophyll *b* (mg 100 g^−1^ FW)	Chlorophyll *ab* (mg 100 g^−1^ FW)	Betacyanin (µg 100 g^−1^ FW)	Betaxanthin (µg 100 g^−1^ FW)	Betalain (µg 100 g^−1^ FW)	Total carotenoids (µg g^−1^ FW)
DS25	24.19 ± 0.04j	10.45 ± 0.08j	34.66 ± 0.15h	26.23 ± 0.11k	27.68 ± 0.15k	53.92 ± 0.42k	562.78 ± 1.15n
DS26	50.86 ± 0.08a	23.49 ± 0.08c	74.37 ± 0.13a	48.67 ± 0.14b	49.59 ± 0.16b	98.28 ± 0.15b	761.41 ± 0.43l
DS27	25.59 ± 0.08h	8.45 ± 0.08k	34.06 ± 0.16i	25.17 ± 0.15l	24.89 ± 0.24l	50.07 ± 0.18l	1451.89 ± 1.25f
DS28	17.89 ± 0.09o	7.61 ± 0.08l	25.52 ± 0.13m	30.44 ± 0.18i	31.42 ± 0.21i	61.87 ± 0.28i	1560.27 ± 1.29d
DS29	12.25 ± 0.04q	6.59 ± 0.04m	18.86 ± 0.12o	23.66 ± 0.14o	24.24 ± 0.17m	47.91 ± 0.42n	1175.19 ± 1.42j
DS30	42.97 ± 0.09c	23.98 ± 0.05b	66.98 ± 0.11c	53.36 ± 0.18a	55.24 ± 0.15a	108.60 ± 0.26a	469.29 ± 1.58o
DS31	25.27 ± 0.07i	5.67 ± 0.08n	30.98 ±0.14k	34.65 ± 0.34e	37.27 ± 0.16d	71.93 ± 0.51e	1587.20 ± 1.29b
DS32	13.35 ± 0.06p	6.62 ± 0.07m	19.99 ± 0.21n	15.42 ± 0.16q	17.27 ± 0.19o	32.70 ± 0.62p	1567.93 ± 1.25c
DS33	34.61 ± 0.02e	18.64 ± 0.05f	53.27 ± 0.13d	33.50 ± 0.34g	32.57 ± 0.17h	66.09 ± 0.26h	1458.13 ± 1.82e
DS34	43.57 ± 0.07b	27.38 ± 0.03a	70.97 ± 0.12b	34.19 ± 0.19f	34.82 ± 0.24f	69.02 ± 0.31f	755.01 ± 1.52m
DS35	20.87 ± 0.08n	5.87 ± 0.06n	26.77 ± 0.13l	17.59 ± 0.28p	17.60 ± 0.28n	35.20 ± 0.28o	1675.38 ± 1.29a
DS36	29.60 ± 0.05f	17.23 ± 0.07g	46.87 ± 0.14f	33.25 ± 0.24h	33.55 ± 0.24g	66.81 ± 0.42g	1342.62 ± 1.65h
DS37	36.28 ± 0.06d	12.50 ± 0.05i	48.80 ± 0.18e	35.52 ± 0.21d	36.76 ± 0.16e	72.29 ± 0.24d	1354.02 ± 1.62g
DS38	22.14 ± 0.09l	21.40 ± 0.04e	43.55 ± 0.19g	24.67 ± 0.42m	24.85 ± 0.22l	49.53 ± 0.24l	1672.97 ± 1.22a
DS39	29.08 ± 0.06g	14.38 ± 0.08h	43.16 ± 0.14g	30.16 ± 0.28j	30.67 ± 0.28j	60.51 ± 0.35j	1194.80 ± 1.05i
DS40	20.89 ± 0.08m	22.52 ± 0.05d	43.43 ± 0.18g	38.25 ± 0.42c	37.49 ± 0.18c	75.76 ± 0.35c	892.04 ± 1.25k
DS41	22.56 ± 0.04k	10.27 ± 0.06j	32.85 ± 0.17j	24.35 ± 0.16n	24.86 ± 0.19l	49.22 ± 0.74m	1672.89 ± 1.26a
Mean	27.76	14.30	42.06	31.12	31.81	62.92	1244.34
CV%	3.3542	1.1285	2.6532	2.6358	1.3284	3.4587	4.3265

CV, Coefficient of variation; n = 6; **Significant at 1% level, Different letters in each columns are differed significantly by Tukey’s HSD test.

Betacyanin ranged from 15.42 to 53.36 µg 100 g^−1^ with a mean value of 31.12 µg 100 g^−1^. Betaxanthin content showed the significant and notable differences in 17 stem amaranth genotypes (17.27 to 55.24 µg 100 g^−1^). High betaxanthin content was observed in four genotypes. Eight genotypes had higher betaxanthin content than the mean value. Betalain ranged from 32.70 to 108.60 µg 100 g^−1^. High betalain content was observed in five genotypes. Eight genotypes had higher betalain content than average value. The range of total carotenoids content was 469.29 µg g^−1^ to 1675.38 µg g^−1^. Three genotypes showed the highest total carotenoids content. Similarly, high total carotenoids were found in four genotypes. Ten genotypes had higher total carotenoids than average value. In this study, we found a significant amount of betacyanin (31.12 µg 100 g^−1^), betaxanthin (31.81 µg 100 g^−1^), betalain (62.92 µg 100 g^−1^) and total carotenoids (1675.38 µg g^−1^) in the stem amaranth. Khanam *et al*.^33^ reported corroborative results for betacyanin, betaxanthin, betalain and total carotenoids content of *A. tricolor*.

### Antioxidant phytochemicals

Table 4 represents TAC, vitamins, TPC, and TFC of stem amaranth. The range of beta-carotene content was 355.35 µg g^−1^ to 1289.26 µg g^−1^. Four genotypes showed high beta-carotene. Ten genotypes had higher beta-carotene than average beta-carotene. The range of vitamin C content was 431.14 to 431.22 µg g^−1^ with a mean value of 746.58 µg g^−1^. Seven genotypes had higher vitamin C than average vitamin C. Vitamin C content was high in four genotypes. The range of total polyphenol content (TPC) was 78.22 GAE µg g^−1^ DW to 228.66 GAE µg g^−1^ DW with a mean value of 156.25 GAE µg g^−1^ DW. Five genotypes showed high polyphenol content. Ten genotypes showed higher polyphenol than average polyphenol content. Prominent variations were noted in the TFC content of stem amaranth genotypes, with a range of 65.89 RE µg g^−1^ DW to 157.42 RE µg g^−1^ DW. The mean value of TFC was 105.84 RE µg g^−1^ DW. TFC showed the following order: DS30 > DS26 > DS40 > DS35 > DS34. Eight genotypes showed higher TFC value than average TFC. The range of TAC (DPPH) was 8.94 TEAC µg g^−1^ DW to 26.61 TEAC µg g^−1^ DW. Five genotypes had high TAC (DPPH). Seven genotypes exhibited higher TAC (DPPH) than average value. The range of TAC (ABTS^+^) was 16.71 TEAC µg g^−1^ DW to 51.73 TEAC µg g^−1^ DW. Five genotypes exhibited high TAC (ABTS^+^) with a mean value of TAC (ABTS^+^) of 30.92 TEAC µg g^−1^ DW. Seven genotypes exhibited higher TAC (ABTS^+^) than average TAC (ABTS^+^).

**Table 4:** Mean performance for betacarotene, vitamin C, TPC, TFC, TAC (DPPH) and TAC (ABTS^+^) of 17 stem amaranth genotypes.

Genotypes	Beta-carotene (µg g^−1^ FW)	Vitamin C (µg g^−1^ FW)	TPC (GAE µg g^−1^ DW)	TFC (RE µg g^−1^ DW)	TAC (DPPH) (TEAC µg g^−1^ DW)	TAC (ABTS^+^) (TEAC µg g^−1^ DW)
DS25	426.45 ± 1.26n	1355.46 ± 2.44a	123.83 ± 0.32m	85.34 ± 0.24k	15.25 ± 0.12f	29.50 ± 0.05f
DS26	578.26 ± 1.26m	739.33 ± 2.06g	156.96 ± 0.42i	155.41 ± 0.25b	25.24 ± 0.15b	45.17 ± 0.11b
DS27	1105.62 ± 1.17g	862.28 ± 2.86d	146.35 ± 0.58j	95.77 ± 0.25j	12.78 ± 0.13h	25.89 ± 0.05h
DS28	1187.28 ± 1.26e	801.34 ± 3.25f	156.46 ± 0.46i	85.25 ± 0.28k	9.21 ± 0.13k	18.21 ± 0.06j
DS29	894.44 ± 1.85k	616.12 ± 2.46i	162.41 ± 0.85h	108.31 ± 0.24h	11.25 ± 0.16j	21.83 ± 0.04i
DS30	355.35 ± 1.88o	616.26 ± 2.48i	195.54 ± 0.92b	157.42 ± 0.16a	26.56 ± 0.11a	49.64 ± 0.04a
DS31	1208.52 ± 1.02d	985.44 ± 2.42b	125.82 ± 0.35l	104.98 ± 0.25i	20.11 ± 0.15c	37.59 ± 0.08c
DS32	1207.55 ± 1.19d	887.24 ± 3.55c	78.22 ± 0.35o	65.89 ± 0.35n	16.28 ± 0.17e	30.43 ± 0.07e
DS33	1116.45 ± 1.35f	431.14 ± 2.28k	146.26 ± 0.23j	68.02 ± 0.36m	8.94 ± 0.21l	16.71 ± 0.06k
DS34	576.43 ± 1.22m	616.28 ± 2.54i	173.54 ± 0.38e	125.71 ± 0.42e	21.61 ± 0.11c	40.39 ± 0.06c
DS35	1289.26 ± 2.05a	369.47 ± 1.45l	168.71 ± 0.52g	143.28 ± 0.24d	12.47 ± 0.14i	23.31 ± 0.09i
DS36	1013.40 ± 1.65i	554.43 ± 1.29j	184.29 ± 0.36c	122.64 ± 0.25f	16.82 ± 0.20e	31.44 ± 0.03e
DS37	1037.28 ± 1.35hh	554.43 ± 1.29j	119.45 ± 0.27n	64.41 ± 0.48o	11.54 ± 0.24j	21.57 ± 0.05i
DS38	1271.46 ± 1.85c	677.51 ± 2.45h	176.22 ± 0.46d	84.77 ± 0.16k	14.55 ± 0.26g	27.19 ± 0.02g
DS39	909.35 ± 1.88j	838.84 ± 2.56e	170.26 ± 0.16f	111.14 ± 0.34g	18.85 ± 0.16d	35.23 ± 0.07d
DS40	680.64 ± 1.34l	1355.14 ± 1.38a	228.66 ± 0.42a	144.55 ± 0.36c	26.61 ± 0.16a	51.73 ± 0.03a
DS41	1275.20 ± 1.39b	431.22 ± 2.56k	143.20 ± 0.32k	76.37 ± 0.35l	10.58 ± 0.18j	19.77 ± 0.02j
Mean	949.00	746.58	156.25	105.84	16.39	30.92
CV%	3.4853	1.3258	1.7568	0.4326	0.3254	0.3524

CV, Coefficient of variation; TAC = Total antioxidant capacity, TPC = Total polyphenol content, TFC = Total flavonoid content, n = 6; **Significant at 1% level, Different letters in each columns are differed significantly by Tukey’s HSD test.

In this study, we found a significant amount of beta-carotene (1289.26 µg g^−1^), vitamin C (1355.14 µg g^−1^) in the stem amaranth, which was relatively higher than *A. tricolor*^3^ of our earlier studies. Our obtained TPC (228.66 GAE µg g^−1^ FW) was higher than the TPC of *A. tricolor* reported by Khanam *et al*.^33^. Our observed TFC (157.42 RE µg g^−1^ DW), TAC (DPPH) (26.61 TEAC µg g^−1^ DW), and TAC (ABTS^+^) (51.73 TEAC µg g^−1^ DW) were corroborative to the results of *A. tricolor* of Khanam *et al*.^33^. The genotype DS40 showed high phenolics and vitamin antioxidants along with high TAC. Similarly, genotypes, DS30 and DS26 had high phenolics, minerals, and antioxidants along with high TAC. These three genotypes could be used as antioxidant profile enriched high-yielding varieties. The high and moderate antioxidant profile enriched genotypes could be used as parents for a future breeding program to generate high-yieldng and antioxidant potential varieties. The present investigation revealed that it is a good source of proximate and minerals, antioxidant leaf pigments, vitamins, and phenolics antioxidants offered huge prospects for feeding the mineral, vitamin, and antioxidant deficient community.

### Correlation studies

Correlations of phytochemicals, antioxidant pigments, and antioxidant potential of stem amaranth are shown in Table 5. The correlation coefficients shown in Table 5 had encouraging findings. We observed a significant positive correlation among TAC (DPPH), chlorophyll *ab*, betacyanin, chlorophyll *a*, betaxanthin, betalain, TAC (ABTS^+^), chlorophyll *b*, and TFC. Shukla *et al*.^34^ also reported positive associations in their earlier work in *A. tricolor*. Similarly, betacyanin, betaxanthin, and betalain showed positive and significant interrelationship among each of them and with TAC (ABTS^+^), chlorophylls, TFC, TAC (DPPH), and TPC which was corroborated with the results of our earlier studies in amaranth^8,9,20–24^ indicating increase in any pigment was directly related to increment of another pigment. The positive and significant interrelationship of TAC (DPPH), pigments, TFC, TPC, and TAC (ABTS^+^) indicated that pigments, TFC, and TPC exhibited strong antioxidant potential. The significant negative association was observed between pigments *vs*. total carotenoids and pigments *vs*. beta-carotene, while total carotenoids and beta-carotene exhibited a significant positive association with TAC (ABTS^+^), TAC (DPPH), TPC, and TFC which was corroborated with the results of our earlier studies in amaranth^20–24^. It indicated that the increment of any leaf pigment had a direct decrement of total carotenoids and beta-carotene. Beta-carotene and total carotenoids exhibited strong antioxidant potential as these traits had significantly and positively associated with TAC (ABTS^+^), TAC (DPPH), TPC, and TFC. There were positive associations between beta-carotene and total carotenoids. In contrast, the negligible insignificant association was observed between vitamin C and all the leaf pigments. Jimenez-Aguilar and Grusak^29^ reported negligible insignificant association for ascorbic acid in amaranth. Whereas, vitamin C was positively and significantly correlated with TAC (ABTS^+^), TAC (DPPH), TPC, and TFC indicating the strong contribution of vitamin C of stem amaranth to antioxidant activity. TAC (ABTS^+^), TAC (DPPH), TPC, and TFC associated significantly and positively among each other, as well as vitamins and pigments, indicated that vitamins, flavonoids, pigments, phenolics strongly contributed to the antioxidant activity of amaranth. In the present investigation, it revealed that leaf pigments, vitamins, phenolics, flavonoids played a significant contribution to the antioxidant capacity of stem amaranth.

**Table 5:** The **c**orrelation coefficient for antioxidant leaf pigments, beta-carotene, vitamin C, TPC, TFC, TAC (DPPH) and TAC (ABTS^+^) in17 stem amaranth genotypes.

Traits	Chl *b* (mg 100 g^−1^ FW)	Chl *ab* (mg 100 g^−1^ FW)	Beta cyanin (µg 100 g^−1^ FW)	Beta xanthin (µg 100 g^−1^ FW)	Betalain (µg 100 g^−1^ FW)	Total catonenoirds (µg g^−1^ FW)	Beta carotene (µg g^−1^ FW)	Vitamin C (µg g^−1^ FW)	TPC (GAE µg g^−1^ DW)	TFC (RE µg g^−1^ DW)	TAC (TEAC µg g^−1^ DW)	TAC (ABTS^+^) (TEAC µg g^−1^ DW)
Chlorophyll *a* (mg 100 g^−1^ FW)	0.75**	0.82**	0.76**	0.78**	0.77**	−0.56**	−0.48**	−0.02	0.75**	0.64**	0.58**	0.83**
Chlorophyll *b* (mg 100 g^−1^ FW)		0.86**	0.80**	0.75**	0.72**	−0.72**	−0.67**	−0.04	0.74**	0.65**	0.63**	0.67**
Chlorophyll *ab* (mg 100 g^−1^ FW)			0.82**	0.74**	0.84**	−0.77**	−0.66**	−0.04	0.77**	0.46**	0.77**	0.83**
Betacyanin (µg 100 g^−1^ FW)				0.88**	0.89**	−0.79**	−0.69**	−0.13	0.73**	0.65**	0.71**	0.78**
Betaxanthin (µg 100 g^−1^ FW)					0.87**	−0.76**	−0.72**	−0.14	0.71**	0.64**	0.70**	0.78**
Betalain (µg 100 g^−1^ FW)						−0.87**	−0.73**	−0.16	0.72**	0.74**	0.71**	0.85**
Total catonenoirds (µg g^−1^ FW)							0.88**	−0.16	0.84**	0.68**	0.78**	0.95**
Betacarotene (µg g^−1^ FW)								−0.15	0.69*	0.74**	0.67**	0.64**
Vitamin C (µg g^−1^ FW)									0.68**	0.65**	0.69**	0.76**
TPC (GAE µg g^−1^ DW)										0.78**	0.76**	0.96**
TFC (RE µg g^−1^ DW)											0.84**	0.89**
TAC (DPPH) (TEAC µg g^−1^ DW)												0.95**

Chl *a*, Chlorophyll *a;* Chl *ab*, Chlorophyl *ab*; TAC, Total antioxidant capacity; TPC, Total polyphenol content; TFC, Total flavonoid content; **Significant at 1% level.

In conclusion, stem amaranth leaves were good sources of potassium, calcium, magnesium, iron, manganese, copper, zinc, chlorophylls, vitamin C, betacyanin, betaxanthin, TAC, betalain, carotenoids, betacarotene, protein, dietary fiber, TPC, carbohydrates, and TFC. It could be used as a leafy vegetable for potential sources of antioxidant leaf pigments, betacarotene, vitamin C, phenolics, minerals and proximate, flavonoids in the human diet for attaining nutritional and antioxidant sufficiency.

## Methods

### Experiment materials, layout, design, and cultural practices

Seventeen stem amaranth genotypes selected from 156 genotypes were sown in Bangabandhu Sheikh Mujibur Rahman Agricultural University, Gazipur, in a randomized complete block design (RCBD) with three replications. It is consumed as a leafy vegetable in the early stage (30 days old). In the later stage (up to 4 months) only stems were eaten as vegetables in different curries which tend to be less nutritious. The experimental unit was 1 m × 1 m. Stem amaranth genotype was grown maintaining the distance of 20 cm between rows and 5 cm between plants. The experimental site was located in the center of the Madhupur Tract (AEZ 28), about 24°23′N 90°08′E, with a mean elevation of 8.4 msl. The site falls under the subtropical zone and has mean temperatures of 29 °C (summer) and 18 °C (winter). There was no precipitation during the cropping season. The experimental field was a high land having silty clay soil. The soil was slightly acidic (pH 6.4) and low in organic matter (0.87%), total N (0.09%) and exchangeable K (0.13 cmol/kg). The soil S content was at par with a critical level, while P and Zn contents were above the critical level (Critical levels of P, S, and Zn were 14, 14 and 0.2 mg kg^−1^, respectively and that of K was 0.2 cmol kg^−1^). During land preparation total compost (10 ton/ha) was applied. We applied recommended fertilizer doses, such as Urea, triple super phosphate, murate of potash and gypsum at 200, 100, 150, and 30 kg/ha, respectively. Thinning was done to maintain appropriate spacing between plants of a row. As a necessity, weeding and hoeing were done at 7 days interval to control the weeds. Proper irrigations were provided to maintain the normal growth of the crop. Leaf samples were collected 30 days after the sowing of seed.

### Chemicals

Solvent: methanol, ethanol, and acetone. Reagents: dithiothreitol (DTT), HNO_3_, standard compounds of pure Trolox (6-hydroxy-2,5,7,8-tetramethyl-chroman-2-carboxylic acid), H_2_O_2,_ potassium persulfate, ascorbic acid, folin-ciocalteu reagent, gallic acid, DPPH (2,2-diphenyl1-picrylhydrazyl), ABTS^+^, rutin, 2, 2-dipyridyl, sodium carbonate, aluminum chloride hexahydrate, and potassium acetate. We bought all solvents and reagents from Kanto Chemical Co. Inc. (Tokyo, Japan) and Merck (Germany).

### Measurement of the composition of proximate

Ash, crude fat, moisture, crude protein contents, fiber, and gross energy were determined through AOAC method^35,36^. Crude protein was estimated through the Micro-Kjeldahl method multiplying nitrogen by 6.25 (AOAC method 976.05). To estimate carbohydrate (g 100 g^−1^ FW), the sum of the percentage of crude protein, ash, crude fat, and moisture was subtracted from 100.

### Estimation of composition of minerals

Stem amaranth leaves were dried at 70 °C for 24 hours in an oven. We ground the dried leaves finely in a mill. The method described by Jimenez-Aguilar and Grusak^29,36^ was used to estimate minerals. Concentrated HNO_3_ was used to digest the samples (250 mg) overnight (room temperature). Then it was set for 2.5 h at 125 °C, followed with 30% H_2_O_2_ for 2 h at 125 °C. The temperature was then increased to 200 °C, and the samples were heated until they were completely dry. After cooling, the samples were resuspended in 15 mL 2% HNO_3_. The following wavelengths (nm): K (404.721), Ca (219.77), Mg (294.20), Fe (262.82), Mn (257.6), Cu (327.39), and (Zn 206.19) were used to determine the concentrations through an inductively coupled plasma optical emission spectroscopy (ICP-OES, Ciros ICP-FCE12, Kleve, Germany). Certified mineral standard was followed to calibrate the ICP-OES daily. Results are expressed in mg and µg per gram of sample dry weight (DW).

### Estimation of carotenoids and chlorophylls

Method of Sarker and Oba^36,37^ was followed to estimate chlorophyll *ab*, chlorophyll *b*, total carotenoids, and chlorophyll *a* through extracting the fresh leaves of stem amaranth in 80% acetone. The absorbance was read at 663 nm for chlorophyll *a*, 646 nm for chlorophyll *b*, and 470 nm for total carotenoids, respectively through a spectrophotometer (Hitachi, U-1800, Tokyo, Japan). Data were expressed as mg chlorophyll per 100 g and µg total carotenoids per g fresh weight.

### Estimation of betaxanthin and betacyanin content

Method of Sarker and Oba^36,38^ was followed to estimate betacyanin and betaxanthin through extracting the leaves of stem amaranth in 80% methyl alcohol having 50 mM ascorbate. Betacyanin and betaxanthin were estimated using a spectrophotometer (Hitachi, U-1800, Tokyo, Japan) at 540 nm for betacyanin and 475 nm for betaxanthin, respectively. The results were expressed as microgram betanin equivalent per 100 gram fresh weight (FW) for betacyanin and micrograms indicaxanthin equivalent per 100 gram FW for betaxanthin.

### Determination of beta-carotene

Beta-carotene content was extracted following the method of Sarker and Oba^36^. 500 mg of fresh leaf sample was ground thoroughly in a mortar and pestle with 10 ml of 80% acetone. After removing the supernatant in a volumetric flask, the extract was centrifuged at 10,000 × g for 3–4 min. The final volume was brought up to 20 ml. The absorbance was taken at 510 nm and 480 nm using a spectrophotometer (Hitachi, U-1800, Tokyo, Japan). Data were expressed as µg beta-carotene per g fresh weight.

The following formula was used to measure the beta-carotene content:

$${\rm{Beta}} \mbox{-} {\rm{carotene}}=7.6\,({\rm{Abs}}.\,{\rm{at}}\,480)-1.49\,({\rm{Abs}}.\,{\rm{at}}\,510)\times {\rm{Final}}\,{\rm{volume}}/(1000\times {\rm{fresh}}\,{\rm{weight}}\,{\rm{of}}\,{\rm{leaf}}\,{\rm{taken}})$$

### Determination of vitamin C

A spectrophotometer was used to measure ascorbate (AsA) and dehydroascorbic acid (DHA) acid from the fresh stem amaranth leaves. Dithiothreitol (DTT) was used for the pre-incubation of the sample and reduction of DHA into AsA. AsA reduced Fe_3_^+^ to Fe_2_^+^. AsA was estimated through measuring Fe_2_^+^ complexes with 2, 2-dipyridyl^36,39^. Finally, the absorbance of the sample solution was read at 525 nm using a spectrophotometer (Hitachi, U-1800, Tokyo, Japan) and data were expressed as µg vitamin C per g fresh weight.

### Extraction of sample for TAC, TFC, and TPC

The leaves were dried in the air in a shade for chemical analysis. 1 g of grounded dried leaves was extracted in 40 ml of 90% aqueous methanol in a tightly capped bottle (100 ml). The bottle was then placed in a shaking water bath (Thomastant T-N22S, Thomas Kagaku Co. Ltd., Japan) for 1 h. The extract was filtered for estimation of total antioxidant capacity, flavonoids, and polyphenols.

### Polyphenols estimation

Method of Sarker and Oba^36,40^ was followed to estimate the total phenolic conten of stem amaranth using the folin-ciocalteu reagent with gallic acid as a standard phenolic compound. Folin-ciocalteu reagent was previously diluted 1:4, reagent: distilled water. In a test tube, 1 ml of diluted folin-ciocalteu was added to 50 µl extract solution and then mixed thoroughly for 3 min. 1 ml of Na_2_CO_3_ (10%) was added to the tube and stand for 1 h in the dark. A Hitachi U1800 spectrophotometer (Hitachi, Tokyo, Japan) was used to read the absorbance at 760 nm. A standard gallic acid graph was made to determine the concentration of phenolics in the extracts. The results are expressed as μg gallic acid equivalent (GAE) g^−1^ DW.

### Flavonoids estimation

The AlCl_3_ colorimetric method^26,36^,^41^ was used to estimate the total flavonoid content of stem amaranth extract. In a test tube, 1.5 ml of methanol was added to 0.1 ml of 10% aluminum chloride, 0.1 ml of 1 M potassium acetate, 2.8 ml of distilled water and 500 µl of leaf extract for 30 min at room temperature. A Hitachi U1800 spectrophotometer (Hitachi, Tokyo, Japan) was used to take the absorbance of the reaction mixture at 415 nm. TFC is expressed as μg rutin equivalent (RE) g^−1^ dry weight (DW) using rutin as the standard compound.

### Assay of antioxidant capacity (TAC)

Diphenyl-picrylhydrazyl (DPPH) radical degradation method^26,36^ was used to estimate the antioxidant activity. In a test tube, 1 ml of 250 µM DPPH solution was added to 10 µl of leaf extract solution (in triplicate) and 4 ml of distilled water and allowed to stand for 30 min in the dark. A Hitachi U1800 spectrophotometer (Hitachi, Tokyo, Japan) was used to read the absorbance at 517 nm. Method of Sarker and Oba^26,36^ was followed for ABTS^+^ assay. 7.4 mM ABTS^+^ solution and 2.6 mM potassium persulfate were used in the stock solutions. The two stock solutions were mixed in equal quantities and allowing them to react for 12 h at room temperature in the dark for preparation of the working solution. Exactly 2850 μl of ABTS^+^ solution (1 ml ABTS^+^ solution mixed with 60 ml methanol) was mixed with 150 μl sample of leaf extract and allowed to react for 2 h in the dark. A Hitachi U1800 spectrophotometer (Hitachi, Tokyo, Japan) was used to read the absorbance against methanol at 734 nm. The percent of inhibition of DPPH and ABTS^+^ relative to the control were used to determine antioxidant activity using the following equation:

$${\rm{Antioxidant}}\,{\rm{activity}}( \% )=({\rm{A}}{\rm{b}}{\rm{s}}.\,{\rm{blank}}-{\rm{Abs}}.\,{\rm{sample}}/{\rm{Abs}}.\,{\rm{blank}})\times 100$$

where, Abs. blank is the absorbance of the control reaction [10 µl methanol for TAC (DPPH), 150 μl methanol for TAC (ABTS^+^) instead of leaf extract] and Abs. sample is the absorbance of the test compound. Trolox was used as the reference standard, and the results were expressed as μg Trolox equivalent g^−1^ DW.

### Statistical analysis

Mineral, chlorophylls, carotenoids, beta-carotene, vitamin C, polyphenols, flavonoids, and antioxidant activity (DPPH & ABTS^+^) analysis were evaluated in three independent samples per replication (each sample was prepared from a combined sample of leaves from multiple plants) and nine samples per genotype. Results were expressed as mean value ± standard deviation per genotype. Every mean represents the average of all measurements for the same genotype (Tables 1–4). ANOVA was performed using Statistix 8 software and the means were compared by Tukey’s HSD test at 1% and level of probability.

### Ethical statement

The lab and field experiment in this study was carried out following guidelines and recommendations of “Biosafety Guidelines of Bangladesh” published by the Ministry of Environment and Forest, Government of the People’s Republic of Bangladesh (2005).

## Data Availability

Data used in this manuscript will be available to the public.
